# Machine learning performance in EEG-based mental workload classification across task types: a systematic review

**DOI:** 10.3389/fnrgo.2025.1621309

**Published:** 2025-09-15

**Authors:** Miloš Pušica, Bogdan Mijović, Maria Chiara Leva, Ivan Gligorijević

**Affiliations:** ^1^mBrainTrain LLC, Belgrade, Serbia; ^2^School of Food Science and Environmental Health, Technological University Dublin, Dublin, Ireland

**Keywords:** mental workload, electroencephalogram (EEG), machine learning, deep learning, pattern recognition, task design, task type, experimental design

## Abstract

The literature features a variety of tasks and methodologies to induce mental workload (MWL) and to assess the performance of MWL estimation models. Because no standardized benchmark task or set of tasks exists, the comparison of different machine learning (ML) solutions in this field is difficult, as their performance is significantly dependent on these factors. In this paper, we present the first comprehensive examination of ML models' performance in EEG-based MWL classification across task types. To achieve this, we categorized ML studies based on the task type used in their experiments and compared models' performances across these categories. Notably, a significant drop in MWL classification accuracy was observed among the best-performing models in multitasking studies where MWL was rated based on quantitative task load, compared to those in single-tasking studies and studies where MWL was subjectively rated. This points to the inherent challenges associated with estimating MWL in more complex tasks such as multitasking. This is particularly relevant for practical applications, as real-world tasks typically involve some degree of multitasking. By comparing ML models' performances across task types, this review provides valuable insights into the state-of-the-art of EEG-based MWL estimation, highlights existing gaps in the field, and points to open questions for further research.

## 1 Introduction

### 1.1 Background

Mental workload is a multifaceted concept that refers to the extent of the cognitive resources utilized by an individual to perform certain tasks. Many cognitive processes contribute to it, including attention, perception, memory, information processing, as well as planning and task switching for more complex tasks and multitasking. It interacts with and overlaps with different factors such as stress, fatigue, motivation, etc. It is influenced by intra individual factors such as cognitive capacity and training, as well as task-specific and environmental factors.

#### 1.1.1 MWL assessment methods and the role of EEG

The complex nature of MWL makes it hard to isolate it from the interfering factors. Besides, there is no universal standard for measuring MWL. Instead, there are different assessment methods that are usually categorized into three main categories: subjective (self-rating questionnaires such as NASA-TLX (NASA Task Load Index)), performance-based (error rates, reaction times, etc.), and physiological methods [Electroencephalography (EEG), Heart Rate Variability (HRV), Pupil Dilation, Functional Magnetic Resonance Imaging (fMRI), Functional Near-Infrared Spectroscopy (fNIRS), etc.].

EEG plays a pivotal role in this field because of its unique advantages over other methods: high temporal resolution (with millisecond precision) and real-time and direct brain monitoring, while being non-invasive and portable, making it well-suited for various environments. Importantly, a notable insight from some studies is that EEG signals inherently contain more information about MWL than other physiological measures (Wilson and Russell, 2003; Coffey et al., 2012; Hogervorst et al., 2014; Aghajani et al., 2017; Liu et al., 2017; Cao et al., 2022). To come to this conclusion, authors developed different machine learning/deep learning (ML/DL) models to classify MWL levels using different physiological signals and compared classification performances. They reported higher accuracies for models trained with EEG data, indicating that EEG encodes more MWL-related information compared to other physiological signals.

#### 1.1.2 EEG correlates of MWL and cognitive mechanisms across task types

Traditionally, EEG spectral features have been used as indicators of MWL, as certain EEG spectral components have been found to correlate with the objective cognitive demands of various tasks. For instance, it has been observed that as task demands increase, frontal theta power (4–7 Hz) increases (So et al., 2017; Pergher et al., 2019), while the parietal alpha power (8–12 Hz) decreases (Pergher et al., 2019). However, despite the correlation demonstrated in many task settings, EEG spectral features lack the necessary precision to differentiate between intermediate MWL levels – those between the lowest and the highest load (Bjegojević et al., 2024). Another issue is that some spectral bands behave differently in different contexts. For example, increased theta band is typically an indicator of a heightened cognitive load, but in some settings it can be an indicator of increased drowsiness (Lin et al., 2008; Awais et al., 2014). However, the same research (Awais et al., 2014) has shown that not all individuals exhibit an increase in theta band, highlighting variability in physiological responses across different people.

Importantly, different tasks at hand engage distinct neural mechanisms and elicit characteristic EEG responses. For example, memory tasks engage mechanisms of maintenance and manipulation of information over short durations. These cognitive demands are associated with increased frontal-midline theta activity in EEG, reflecting enhanced mental effort (Gevins and Smith, 2000). Visual cognitive tasks, including spatial attention and search, involve visuo-spatial attention and top-down control, and are typically marked by alpha-band desynchronization over parieto-occipital regions, particularly contralateral to the attended visual field (Sauseng et al., 2005). Similarly, arithmetic tasks engage frontal-parietal networks for numerical reasoning and mental calculation. Increasing arithmetic difficulty induces stronger theta-band synchronization, particularly over frontal regions (Sammer et al., 2007). On the other hand, multitasking involves handling more than one task simultaneously. It requires splitting attention across multiple tasks and switching between them, introducing specific cognitive challenges. Namely, splitting attention reduces the cognitive capacity available for each task, potentially leading to decreased efficiency, as no single task receives full cognitive focus (Strayer et al., 2017). Additionally, task switching involves extra mental effort to disengage from one task and engage with another, incurring so called “switch costs” that can temporarily impair performance (Monsell, 2003). It heavily relies on cognitive control processes supported by the lateral prefrontal cortex (Dove et al., 2000). These processes are consistently associated with increased frontal midline theta activity, reflecting elevated cognitive control demands during task switching (Cavanagh and Frank, 2014). Also, there is significant variability in how individuals handle multitasking, with differences in efficiency and strategic approaches (Crews and Russ, 2020; Morgan et al., 2013). Unlike the more straightforward nature of single-tasking, multitasking often demands a dynamic and flexible cognitive strategy, introducing a layer of complexity that is not present in single-tasking.

As discussed above, different EEG spectral bands correlate with different aspects of MWL and vary across task types and conditions. Hence, defining a universal EEG-based MWL metric remains a challenge. Literature suggests that integrating information from multiple frequency bands provides a more accurate estimation of MWL than relying on a single band. For example, the ratio of frontal theta and parietal alpha power (Kartali et al., 2019; Pušica et al., 2023), and ratio of beta and alpha power (Caiazzo et al., 2023) have been shown to correlate with objective task load measures for many tasks. These metrics indicate the complex relationships among different spectral bands. However, modeling these relationships with traditional rule-based approaches is challenging.

#### 1.1.3 Machine learning approaches and methodological variability in EEG-based MWL estimation

Machine learning comes as a solution to handling the complexity and variability in EEG data—learning from large datasets to decode the intricate patterns that correlate with MWL. Unlike traditional rule-based metrics (spectral power bands), ML algorithms can adapt to non-linear and context-specific patterns, making them better suited for generalizing across diverse conditions. Numerous models have been used for MWL classification, including classical machine learning methods such as support vector machines, k-nearest neighbors, and random forests, as well as deep learning architectures like convolutional and recurrent neural networks (Zhou et al., 2022). Various EEG preprocessing techniques and feature extraction methods have also been employed (Zhou et al., 2022). Although research in this field is expanding (Demirezen et al., 2024), the methodologies used in the studies vary considerably. According to Demirezen et al., 73% of studies used their own datasets, and they also often exhibit reproducibility issues. ML/DL models for EEG-based MWL estimation are trained and evaluated on different datasets that utilize different tasks to induce MWL. Also, these models vary in terms of robustness against inter-session, inter-subject, and inter-task variations. Besides, data collection varies across studies, employing different number of electrodes, electrode layouts, and signal-to-noise ratios. As a result, performance metrics are challenging to compare due to these key methodological differences.

#### 1.1.4 Research gap and motivation for the review

Among the various sources of methodological variability, one key factor that remains largely unexplored is how task type and design influence the discernibility of MWL levels through EEG. This constitutes a critical gap in the field. Researchers generally overlook this question focusing mainly on achieving high accuracies. To the best of our knowledge, this literature review is the first to address this important question.

The objective of this study is to review and analyze the performance of EEG-based ML/DL models for MWL classification across different task types, highlight some flawed methodologies in the literature, and identify open questions in the field.

### 1.2 Paper structure

The paper is structured as follows:

- Section 2 describes the methods used to conduct this systematic literature review, including the protocol for selecting relevant papers.
- Section 3 provides a statistical overview of the selected papers and presents the categorization of papers.
- Section 4 delves into detailed analysis within the categories of single-tasking, multitasking, and cross-task MWL classification studies. It primarily focuses on evaluation of ML/DL models within these categories with the goal of comparing MWL classification performance across the categories. Additionally, experimental design flaws identified in the literature are systematized in Section 4.3.
- Section 5 concludes the paper by summarizing the main findings, highlighting limitations of the review, and identifying open questions for future research.

## 2 Materials and methods

The review of the state of the art was carried out in a systematic–structured, predefined, and transparent way, minimizing the risk of bias in the paper selection process.

### 2.1 Databases selection

The selection of databases was carefully considered to ensure a comprehensive capture of the relevant literature. Four extensive databases were searched: Web of Science, IEEE Xplore, PubMed, and Scopus. This selection of databases was guided by the objective to compile a diverse and exhaustive set of literature that spans across the technical, engineering, biomedical, psychological, and human factors domains, which are all relevant to the estimation of MWL through EEG using machine learning.

### 2.2 PRISMA protocol and keywords

The literature search and review process were guided by the Preferred Reporting Items for Systematic Reviews and Meta-Analyses (PRISMA) method (Moher et al., 2010). PRISMA is a widely recognized framework that outlines a set of items to include when reporting a systematic review, promoting clarity, transparency, and rigor.

The search strategy was designed to capture papers on MWL estimation from EEG using machine learning. The search key combined EEG terms with a variety MWL descriptors in paper titles and included ML/DL-related terms, along with terms like estimation and classification in the abstracts. The time span for the publication date extended from the earliest recorded date to August 2024. Due to their strong emphasis on indexing only peer-reviewed papers, Web of Science and PubMed were searched for all publications, while IEEE Xplore and Scopus were limited to journal articles. The specific key syntax was dependent on each database, but the semantics was the same. The search term was structured as follows:

Title: (“EEG” OR “Electroencephalography”) AND (“Mental Workload” OR “Cognitive Load” OR “Cognitive Workload” OR “Mental Load” OR “Task Load” OR “Task Demand” OR “Workload”); Title/Abstract: (“Machine Learning” OR “Deep Learning” OR “feature” OR “signature” OR “Neural Network” OR “Transformer” OR “Supervised Learning”) AND (“estimation” OR “classification” OR “assessment” OR “recognition” OR “prediction”).

The inclusion criteria included peer-reviewed papers written only in English language. The exclusion criteria ruled out any papers where:

- The experiment was not described with sufficient clarity to understand how the task difficulty levels were adjusted in the experiment. This was identified as an issue affecting the reproducibility of studies in a review by (Demirezen et al. 2024).
- Numerical results of MWL classification were not provided.
- Results were from the same dataset as already included papers authored by the same research group, unless they provided new insights relevant to the research question.
- The paper was a review paper where experimental design of included papers was not given in detail.

**Figure 1:**
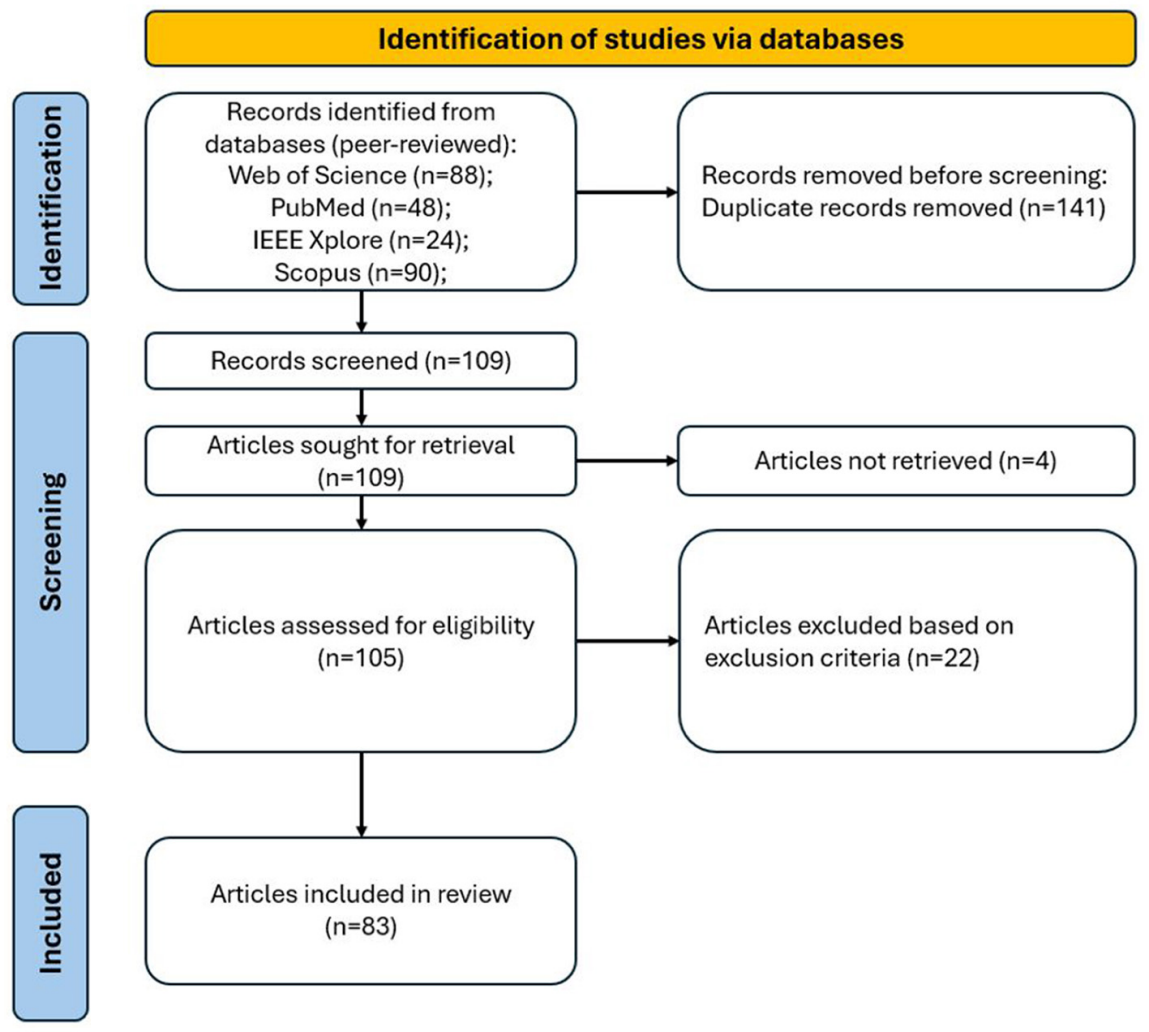
Flow-chart of papers selection.

The systematic literature review flow diagram, guided by the PRISMA methodology, is illustrated in Figure 1. Initially, the search found 250 records. After the removal of duplicate records, 105 records were retrieved and underwent screening based on exclusion criteria. The selection led to 83 articles to be included in the research. These articles underwent a detailed full-text analysis.

## 3 Results

### 3.1 Bibliometric analyses

**Figure 2:**
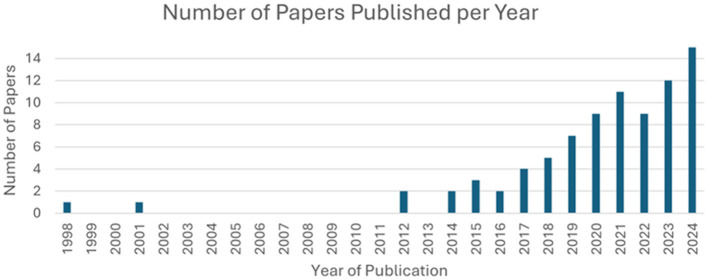
Numbers of publications across years.

A bibliometric analysis was conducted to evaluate the contributions from countries and the number of papers published per year that were included and analyzed in this review. Over 85% (72 out of 83) of the analyzed studies have been conducted since the year 2017. As depicted in Figure 2, publications on this topic show a consistent upward trend over the years.

**Figure 3:**
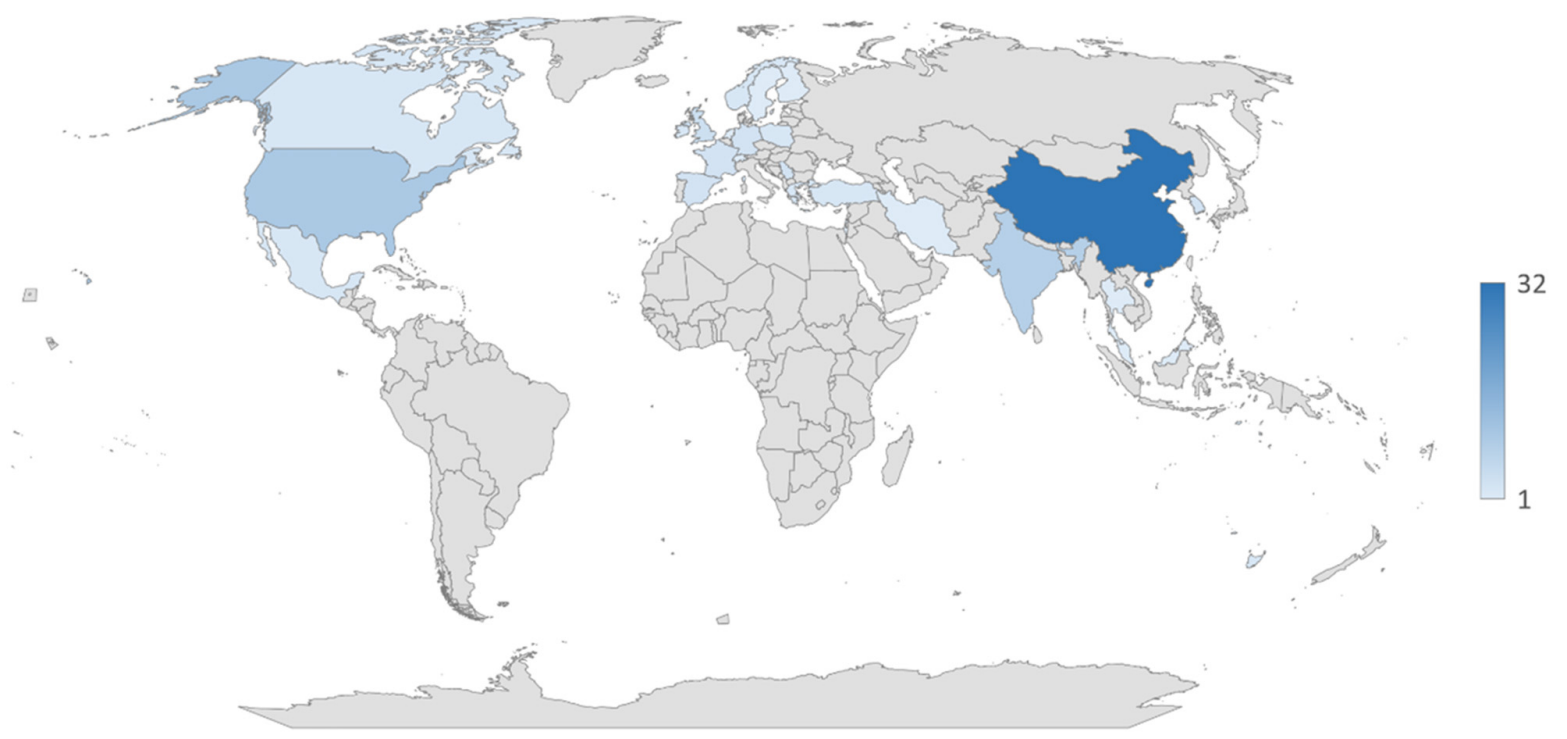
Geographical distribution of published papers.

Figure 3 presents the contributing countries in this field. The highest number of studies (32) were conducted in China, followed by the USA (10), and India (8). The rest of the countries had 5 or less publications per country.

### 3.2 Statistical analysis

**Figure 4:**
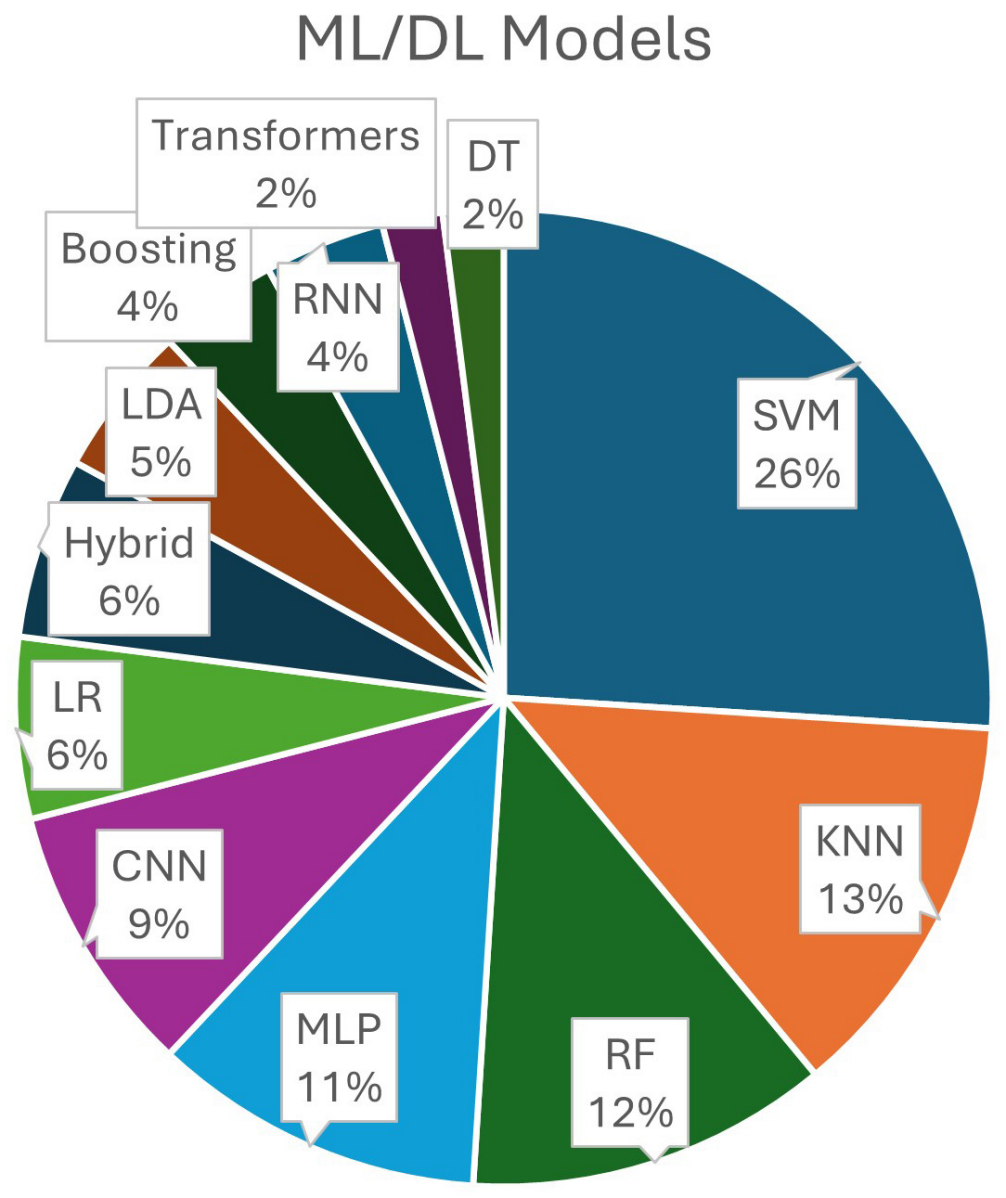
ML/DL models distribution: SVM (Support Vector Machine); KNN (K-Nearest Neighbors); RF (Random Forest); MLP (Multi-Layer Perceptron); CNN (Convolutional NN); LR (Linear/Logistic Regression); Hybrid (hybrid models-combinations of convolutional, recurrent, and transformer models); LDA (Linear Discriminative Analysis); Boosting models; RNN (Recurrent NN); Transformers; DT (Decision Trees).

Distribution of ML/DL models (percentage of analyzed studies that employed certain models) is visually presented in Figure 4.

Classical ML models such as SVMs, KNNs, and RFs are the most frequently used, while deep learning models remain in the minority—indicating that most studies still rely on feature generation for MWL classification.

**Figure 5:**
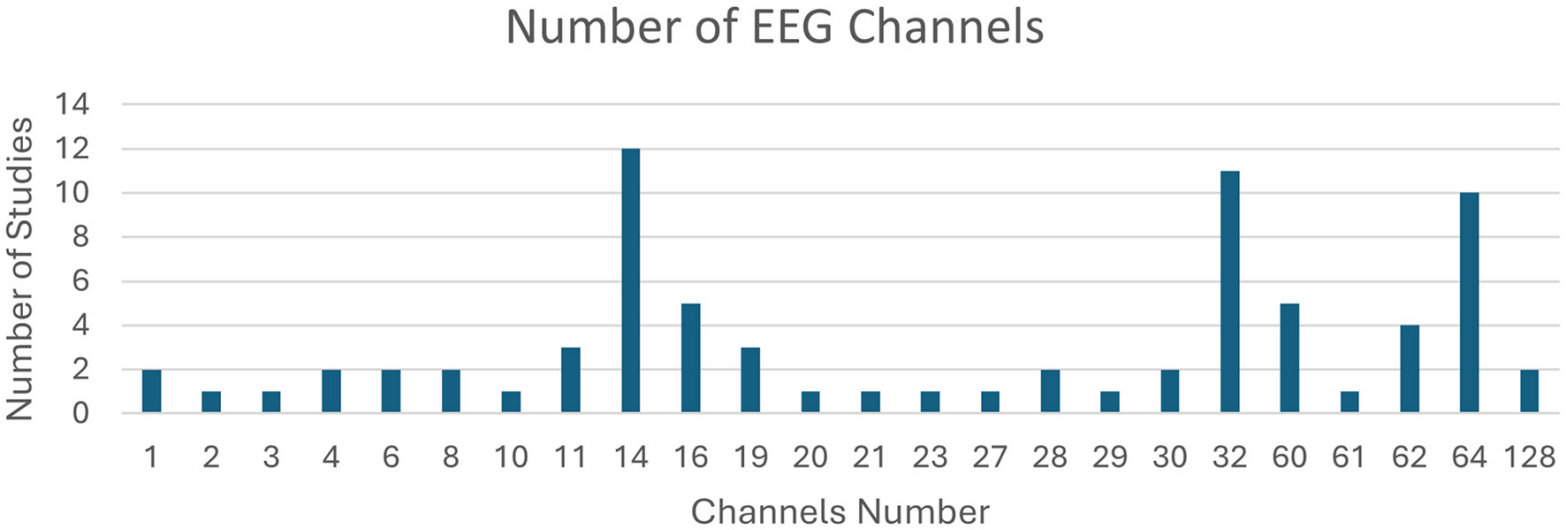
Numbers of EEG channels used in the analyzed papers.

Studies employed different EEG acquisition devices with various layouts. Numbers of channels used across studies are presented in Figure 5.

**Figure 6:**
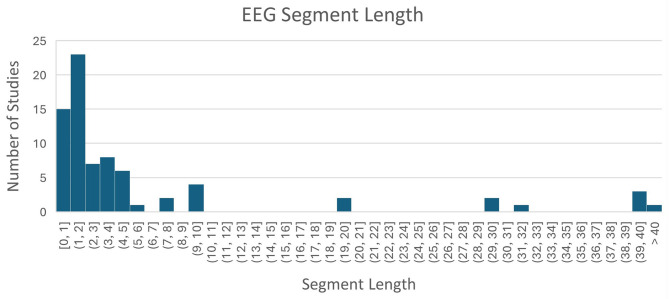
Lengths of EEG segments used in the analyzed papers.

**Figure 7:**
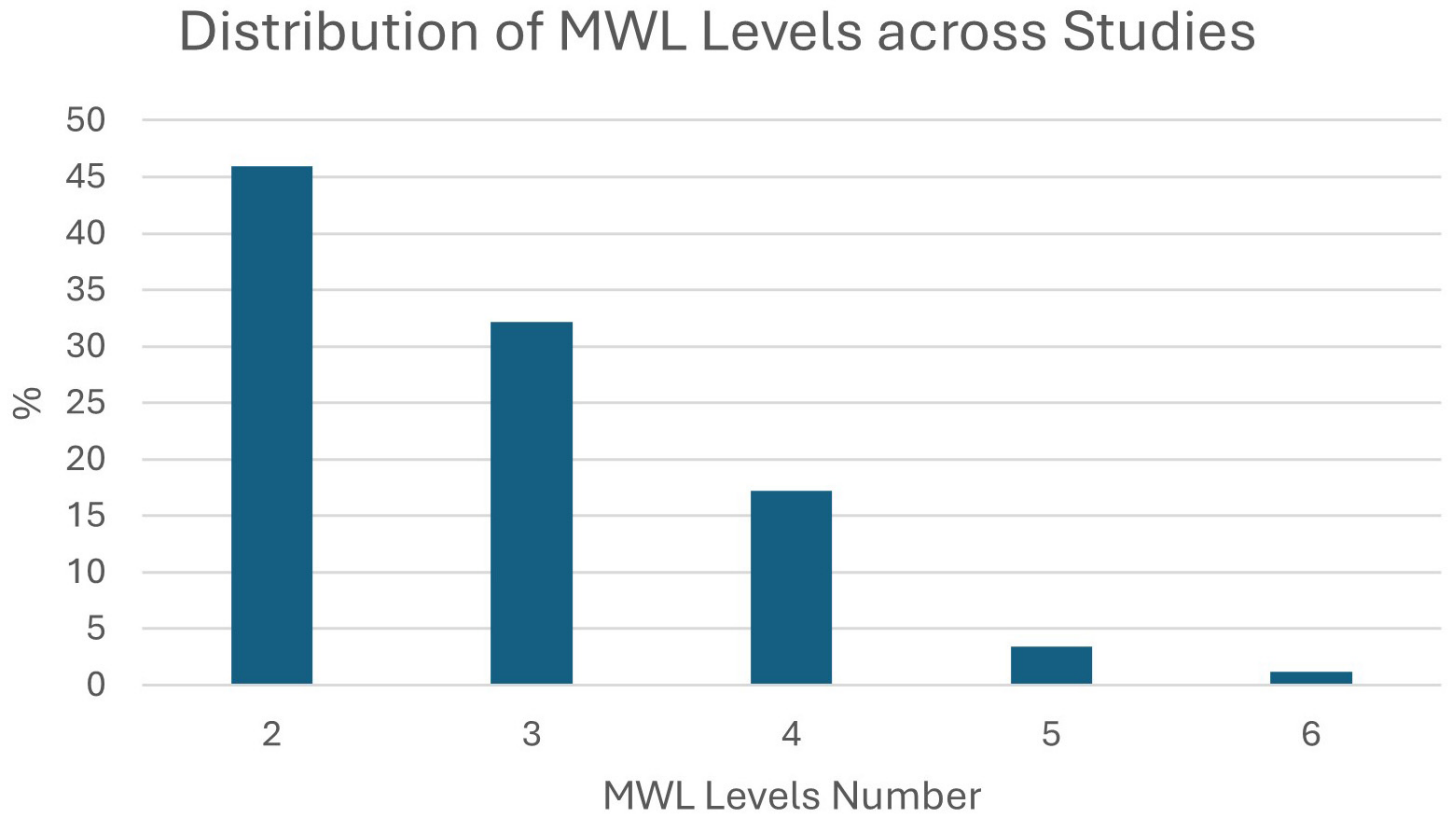
Percentage of studies (out of all included) that used a given number of MWL levels for classification (models' output dimension).

Models classified EEG segments of various lengths and various numbers of classes (MWL levels). As we can see in Figure 6, most studies used EEG segment lengths below 2 s, opting for higher temporal resolution of predictions. Also, the decreasing trend in Figure 7 shows that less studies opted for higher classification resolution (more MWL levels), with the majority of them using only two classes.

### 3.3 Classification of studies

Despite the growing interest in EEG-based MWL estimation in recent years, the field still lacks an established benchmark or theoretical constructs against which different MWL estimation models could be evaluated (Longo and Leva, 2021). There is no standardized dataset or experimental task for benchmarking MWL estimation models, like in other fields of machine learning applications such as image recognition (Deng et al., 2009) or speech recognition (Panayotov et al., 2015). Consequently, researchers do not have standardized protocols or guidelines for the design of experiment for MWL estimation. This practice results in the utilization of a wide variety of tasks in experiments and numerous ways MWL levels are adjusted within experiments. This makes it challenging to consistently and effectively compare the performance of different models across studies.

With this gap in mind, our research focused on examining machine learning models' MWL classification performance for different task types and ways MWL levels were adjusted and rated (labeled) in experiments. To systematically explore this, we categorized the studies based on these factors. Our objective was to analyze how different ML/DL models, as reported in various papers, perform across these categories.

Based on the task type, studies were categorized into two big groups: single-tasking and multitasking. Additionally, a third category was included to analyze papers that examined cross-task MWL estimation—training models on one task and evaluating them on different tasks.

Further division was based on the specific task employed. In the single-tasking category, we identified: memory tasks, arithmetic tasks, multimedia comprehension tasks, visual cognitive tasks, and other tasks. In the multitasking category, we identified more complex tasks including systems control, flight/drive simulation, MATB, and SIMKAP.

Based on how MWL was rated (labeled), we distinguish between:

- task load-based (dependent on the predetermined experimental design)
- subjective MWL-based (dependent on the participants' subjective MWL assessment after completing parts of the task)
- performance-based (dependent on task performance, e.g., error rate)

It is important to say that, even though MWL can be affected by various factors like stress, noise, temperature, interruptions, and distractions, our study specifically concentrated on the impact of task load. Task load can be changed in different ways, depending on the type of the task. In single-tasking scenarios, task load levels are typically manipulated by modifying the complexity of the task (e.g., in memory task–changing the number of symbols to be remembered). On the other hand, multitasking scenarios are different in the sense that task load levels can be modified in various ways—we identified two subcategories:

- Quantitative task load adjustment: Increasing/decreasing frequencies of occurrences of subtasks participants had to manage, keeping the same set of subtasks across different load levels. This adjustment ensures that while participants engage with the same subtasks, subtasks' frequency increases at higher levels, requiring more frequent and rapid interactions.
- Qualitative task load adjustment: Adding/removing subtasks at higher/lower levels. This method introduces additional subtasks at higher levels that are not present at lower levels, thus expanding the set of subtasks that participants must manage as the workload intensifies.
- Invariant task load: Task load remains unchanged across the experiment, hence there are no task load levels. Instead, MWL levels are derived from subjective ratings (subjective perception of the task load across participants).

Table 1 summarizes the categorization of the reviewed papers. The table illustrates where each study fits into the defined categories, providing a clear overview of the research landscape concerning task types, MWL rating methods, and task load adjustment.

**Table 1:** Classification of papers.

**Single/Multi Tasking**	**MWL Rating Method**	**Task Type**	**Task Subtype**	**Publications**	
			*n*-back	Gevins et al., 1998	Grissmann et al., 2020
					Brouwer et al., 2012	Gupta et al., 2021
					Mühl et al., 2014	Cao et al., 2022
					Aghajani et al., 2017	Aksu et al., 2024
					Dai et al., 2017	Beiramvand et al., 2024
					Samima and Sarma, 2019	
		Memory task		Modified *n*-back	Kutafina et al., 2021	Hernández-Sabaté et al., 2022
			Sternberg task		Roy et al., 2016	Jiménez-Guarneros and Gómez-Gil, 2020
					Jiao et al., 2018	Kwak et al., 2020
					Zhang et al., 2019	Zhou et al., 2023
					Qiao and Bi, 2020	Havugimana et al., 2024
Single-tasking	Task load-based			Sarailoo et al., 2022	Yoo et al., 2023
				Mazher et al., 2022	
		Multimedia comprehension			
		Intelligence test		Friedman et al., 2019	
				Yu et al., 2015	Liu Y. et al., 2023
		Visual cognitive task		Ladekar et al., 2021	Pušica et al., 2024a
				Sahin Sadik et al., 2022	Li H. et al., 2024
				Dimitriadis et al., 2015	Sharma et al., 2021
				Spüler et al., 2016	Yedukondalu and Sharma, 2023
		Arithmetic		Plechawska-Wójcik et al., 2019	Yan et al., 2023
				Lu, 2024	Hemakom et al., 2024
				Ramaswamy et al., 2021	
	Performance-based			Xiong et al., 2020	
**Single/Multi Tasking**	**MWL Rating Method**	**Task Load Adjustment Method**	**Task Subtype**	**Publications**	
			MATB	Zhou et al., 2023	Chen Y. et al., 2024
			Systems control	Yin and Zhang, 2017	Yang et al., 2019
		Qualitative task load adjustment: adding/removing subtasks			Tao et al., 2019	
					Ling et al., 2001	Zanetti et al., 2022
	Task load-based			Blanco et al., 2018	Pei et al., 2021
			Flight/drive simulation	Dehais et al., 2019	Liu X. et al., 2023
				Becerra-Sánchez et al., 2020	Wang Y. et al., 2024
			Memory and typing	Chiang et al., 2023	
Multitasking		Quantitative task load adjustment: increasing/decreasing frequency of subtasks	MATB	Chandra et al., 2015	Qu et al., 2022
					Hefron et al., 2018	Ke et al., 2023
					Salaken et al., 2020	Salvan et al., 2023
				Qu et al., 2020	Yan et al., 2023
				Ke et al., 2021	Jin et al., 2024
				Albuquerque et al., 2022	Pušica et al., 2024b
			Systems control	Li H. et al., 2024	
			Flight/drive simulation	Fan et al., 2018	
				Das Chakladar et al., 2020	Shao et al., 2024
				Zhu et al., 2021	Yan et al., 2023,
	Subjective MWL	Invariant task load	SIMKAP	Fan et al., 2022	Wang Z. et al., 2024
				Raufi and Longo, 2022	Raufi and Longo, 2024
				Yedukondalu and Sharma, 2023	
			**MWL rating method**	**Publications**	
				Baldwin and Penaranda, 2012 (memory ↔ memory ↔ memory),	Cabañero Gómez et al., 2021 (memory ↔ Stroop)
				Ke et al., 2015 (memory ↔ memory)	Hernández-Sabaté et al., 2022 (memory ↔ flight simulation)
Cross-task studies			Task load-based	Dimitrakopoulos et al., 2017 (memory ↔ arithmetic)	Guan et al., 2023 (memory ↔ memory)
				Zhang et al., 2019 (memory ↔ arithmetic)	Yin Z. et al., 2024 (SIMKAP ↔ ACAMS ↔ arithmetic)
				Kakkos et al., 2021 (memory ↔ arithmetic)	Chen J. et al., 2024 (MATB ↔ memory)

Several task names are abbreviated in the table (MATB: Multi-Attribute Task Battery, ACAMS: Automation-enhanced Cabin Air Management System, SIMKAP: Simultaneous Capacity, etc.). Readers are referred to the original studies for detailed descriptions of each task.

**Figure 8:**
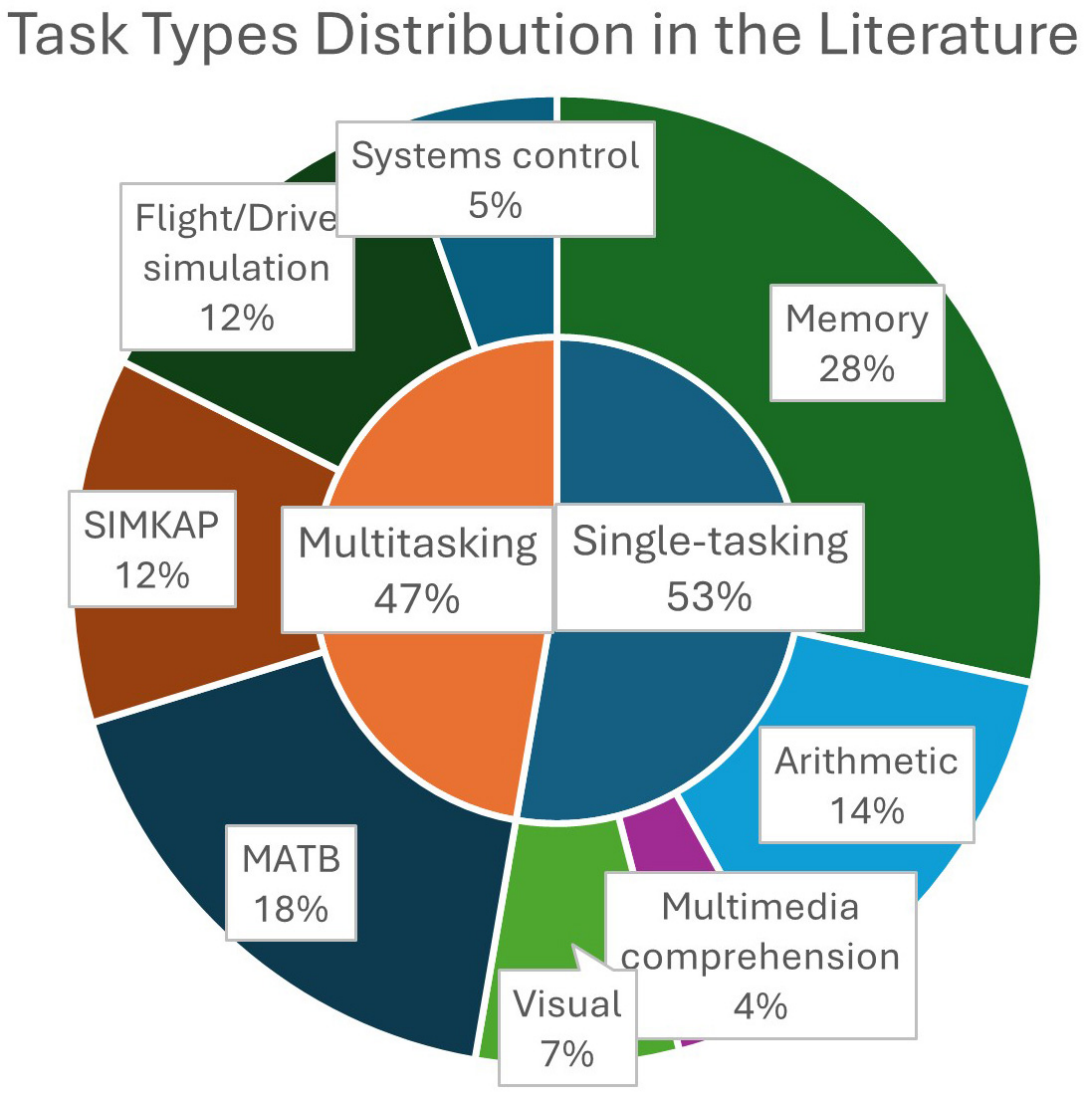
Distribution of task types among analyzed papers.

Distribution of task types and subtypes in the review is visually presented in Figure 8.

## 4 Discussion

### 4.1 Evaluation criteria for studies

In the following Sections, EEG-based MWL classification within the above defined categories will be discussed. The primary criterion for evaluating these models will be their classification accuracy. However, other important characteristics will also be discussed to provide a comprehensive overview of models' performance:

- Number of MWL levels: A higher number of levels enhances the granularity of model predictions but also makes classification more challenging, potentially reducing accuracy (Moral et al., 2022).
- EEG segment length: Defines the model's temporal resolution, with shorter segments providing higher resolution. However, too short segments may not capture sufficient information for accurate MWL classification, potentially reducing accuracy. The optimal length should be determined based on the estimated dynamics of MWL fluctuations throughout the experiment (Maarouf et al., 2024).
- Model robustness: This indicates the model's performance consistency across sessions, subjects, or tasks—a key feature for practical applicability, with higher robustness allowing for broader usability. We define the following robustness levels in ascending order, from least to most robust:

° Random data split model training/testing: This method randomly distributes EEG segments between training and test sets. The problem with this training approach is data leakage, as segments from the same session or close temporal proximity may end up in both the training and test sets, leading to overestimated performance results and compromising its ability to generalize [Brookshire et al., 2024; Yin C. et al., 2024 (preprint)].
° Cross-session robustness (session-independent training/testing): Indicates the model's ability to maintain performance across different sessions for the same subject. It indicates robustness against variations that occur in different experimental sessions, like intrapersonal states such as fatigue, stress or circadian rhythm, slight changes in sensor placement or environmental conditions. Studies that addressed this problem have had participants perform multiple experiment sessions—the sessions used in the test set were not used in the training set.
° Cross-subject robustness (subject-independent training/testing): In this case, a model is trained to generalize across individuals, meaning that it predicts MWL without being tailored to the unique characteristics of a particular subject. The test set consists of EEG data from individuals excluded from training.
° Cross-task robustness: The model is trained on one task but tested on a different one. Making model effective across various types of tasks is particularly valuable for real-world applications where the model needs to handle diverse cognitive demands without retraining for each new task type. However, achieving this level of robustness is particularly hard due to substantial differences in brain activation patterns across tasks, which may not be interpretable or detectable by a single model (Penaranda and Baldwin, 2012; Walter et al., 2013). Furthermore, tasks differ in cognitive load dimensions, such as memory, attention, problem-solving, and motor skills—each influencing distinct neural activations. Adapting a model to generalize across such diverse conditions presents a major challenge in MWL estimation today.

**Figure 9:**
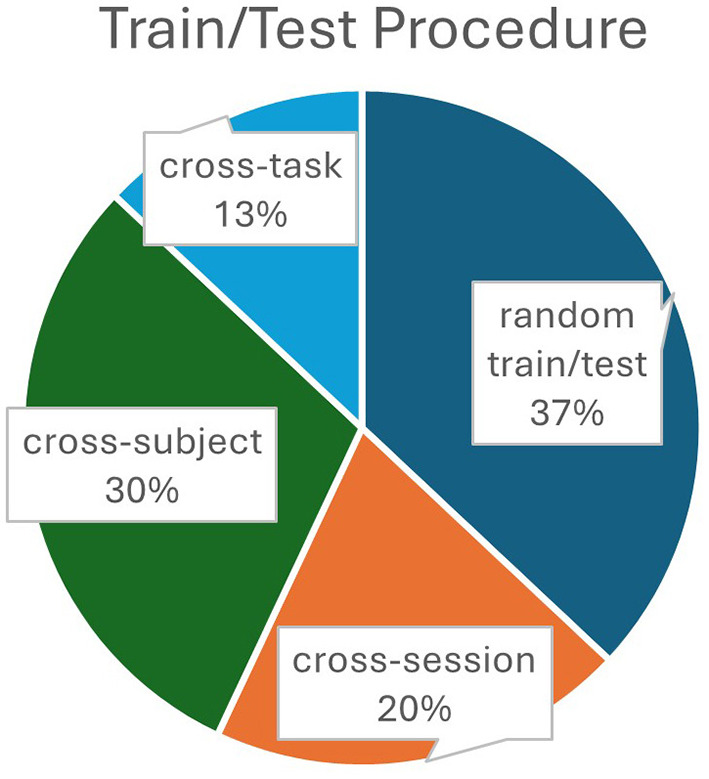
Percentages of the training/testing procedures used in the analyzed studies.

Percentages of the training/testing procedures used in the analyzed studies are visually presented in Figure 9, showing that a significant proportion of studies (37%) employed the random data split model training/testing procedure which is susceptible to data leakage issue explained above.

The subsequent Sections will concentrate on analyzing the categories of studies listed in Table 1, evaluating them based on the models' performance criteria and characteristics given above.

### 4.2 Assessing MWL classification across different task categories

This Section delves into the assessment of MWL classification across various task categories, with a primary focus on differentiating between single-tasking and multitasking settings, along with a separate category of studies addressing cross-task MWL classification. The first two categories inherently impose different task management approaches: single-tasking allows individuals to focus on one task at a time, while multitasking requires frequent attention splitting and task switching, which potentially triggers distinct MWL-related EEG patterns.

Our focus will be on task load levels that remain within the cognitive limits of individuals. The main reason for this is that in scenarios where many errors occur (overload state), it is difficult to ascertain the participants' MWL, as they are no longer adhering to the task requirements. Another reason is that we are interested in manageable task loads, concentrating on realistic and sustainable work. This approach enhances the applicability of our findings: in practical scenarios, it is important that MWL monitoring and management systems can recognize and respond to incremental changes in workload (Lagomarsino et al., 2022). This capability ensures that interventions or adjustments can be made before an individual reaches a state of overload, promoting efficiency and preventing burnout.

#### 4.2.1 Single-tasking

Single-tasking refers to task management in which an individual concentrates on one task at a time, allowing for sustained and focused cognitive engagement. These tasks can be further categorized based on their specific type. Categories we will discuss include memory tasks, arithmetic tasks, multimedia comprehension tasks, visual cognitive tasks, and other tasks.

##### 4.2.1.1 Overview of single-tasking studies

###### 4.2.1.1.1 Memory tasks

This is the most numerous category, including *n*-back tasks, modified *n*-back tasks and Sternberg tasks. These tasks are designed to assess and engage the working memory capabilities of individuals. Participants are presented with a series or group of stimuli (such as letters, numbers, symbols, images, or sounds) and are required to recall the stimulus under varying task load levels. Task load is adjusted by changing task parameters in the following way:

- *n-back*: the “*n*” value, which indicates how many previous stimuli the participant must remember and recognize, can be increased to enhance complexity.
- *Sternberg*: the size of the memory set (the number of items to be memorized) is adjusted.

Performance metrics like accuracy and reaction time are utilized. Importantly, the experiments with these tasks are highly controlled with minimal physical movements. As the task flow in this case was well segmented, with predefined time slots for symbols memorization and recall prompts, as well as other task flow elements, EEG signal window (segment) for classification was positioned around the stimuli presentation time (symbols memorization and recall time). This alignment ensures that the time of the window corresponds to the respective engagement time with the task, matching the MWL levels precisely. As each task trial (memorization and recall) lasted only a few seconds, the EEG signal window length varied from a fraction of a second (0.1–0.5 s) to a few seconds (up to 4 s). Alternatively, some studies (Aghajani et al., 2017; Cao et al., 2022; Hernández-Sabaté et al., 2022) opted for longer window lengths of 20 s or even 40 s, aggregating multiple trials of the same MWL level. However, better classification accuracies were generally achieved with shorter windows, lasting up to a few seconds. Interestingly, the highest reported accuracy (98.18%) was achieved using a subject-independent (cross-subject robust) model with four task load levels (Jiménez-Guarneros and Gómez-Gil, 2020), even though it is generally easier to achieve similar accuracy with less robust models and fewer classification levels. The study employed the Sternberg memory task with four levels, where participants were shown 2, 4, 6, and 8 letters to memorize. It involved 13 participants, using a 3.5 s EEG window length, and a recurrent residual neural network architecture and a domain adaptation method.

###### 4.2.1.1.2 Mental arithmetic tasks

These tasks are designed to test participants‘ ability to perform basic arithmetic operations mentally. Typically, participants are shown a simple arithmetic expression, usually addition or subtraction of two integers and must calculate the answer in their heads and then decide if a presented number matches the solution to the expression. To adjust the task load, task parameters such as the number of digits in the numbers are varied. For instance, the number of digits in numbers can be increased to enhance complexity (e.g., level one could be the addition of two one-digit numbers, level two could be the addition of two two-digit numbers, etc.). As with memory tasks, these arithmetic tasks are conducted under controlled conditions to minimize physical movement. Performance metrics like accuracy and reaction time are utilized. The task flow was structured, with predefined intervals for calculating expressions and other task-related activities. Accordingly, the EEG signal window for classification was aligned with the period of expression calculation, ensuring that the window timing matches the actual engagement time with the task and corresponds accurately with the MWL levels. Typically, these time windows ranged from 0.5 s to 5.5 s. (Dimitriadis et al. 2015) implemented five task load levels of an addition task, where participants added two numbers with varying total digit counts and obtained a high classification accuracy of 96%. However, the model was trained individually for each of 16 participants, and the task trials (EEG segments) were randomly split between training and test sets. This random training/test data partitioning approach led to data leakage, undermining the reliability of the reported accuracy. On the other hand, (Xiong et al. 2020) reported a very high subject-independent classification accuracy (97.2%) classifying two MWL levels based on subjects' error rates (low vs. high error rate) using SVM model. The task consisted of subtraction of two-digit numbers from four-digit numbers.

###### 4.2.1.1.3 Multimedia comprehension tasks

Significant due to their widespread occurrence in everyday life. Namely, audio and visual stimuli are present in both leisure and work activities, ranging from movie watching and video games playing to engaging with educational materials and online meetings. Substantial potential for practical applications include adaptable educational material for optimal knowledge acquisition and student engagement, evaluation of lecture understandability, or assessing the effectiveness of presentation styles or designs. Steps in this direction have been made by Sarailoo et al., 2022, who classified EEG segments from 30 participants watching multimedia educational videos of two categories: videos adhering to optimal educational design principles and those that violated them. An accuracy of 84.5% was achieved using SVM. Similarly, Yoo et al., 2023 classified educational video materials labeled as more and less complicated, attaining an accuracy of 87% for binary classification using a bidirectional Long Short-Term Memory (LSTM) architecture with an attention mechanism. However, the models used in these studies were trained with a random data split between training and test sets, which compromises the robustness necessary for practical relevance.

###### 4.2.1.1.4 Virtual tasks

Many everyday activities include visual cognition: reading, navigation, visual search, etc. Possible applications include optimizing user interface designs and improving human-computer interaction by adapting visual complexity to cognitive capacity. An interesting task designed by Li Z. et al. (2024) tested image recognition with different levels of blurriness. The paper reported 88.13% accuracy for subject-independent 3-level classification of 1 s EEG segments. However, the highest accuracy (96%) was reported by (Sahin Sadik et al. 2022), employing a well-known Stroop test (naming colors of the shown words), classifying 5 s EEG segments into 5 levels using subject-independent model training. However, the paper does not specify whether feature selection was performed in subject-independent way or on the full dataset (potential data leakage).

###### 4.2.1.1.5 Other tasks

An experiment that closely mimics a common industry setting was conducted in a simulated manual assembly line environment, where 23 participants assembled hand-sized items while following visual instructions of different complexity (Pušica et al., 2024a). An accuracy of 90.8% was achieved for subject-independent binary classification using CNN model and 10 s EEG segments. It is also worth mentioning a non-standard type of task utilized for MWL assessment in the form of an intelligence test, involving many task load levels. Namely, (Friedman et al. 2019) applied an intelligence test comprising 36 tasks (levels) of increasing difficulty and performed classification using the XGBoost algorithm. They achieved a surprisingly high classification accuracy of 70%, despite the large number of classes. However, similar to the multimedia comprehension studies, they used the same random data split strategy, leading to issues with model robustness that compromised the relevance of the results.

##### 4.2.1.2 Summary of single-tasking studies

In this Section, we have explored various task categories within the single-tasking framework, focusing on tasks' design, different levels of robustness, number of MWL levels, and MWL classification accuracy. Here, we highlight the most relevant solutions across categories, based on their robustness and classification accuracy:

- Memory task: 98.18% accuracy, 4 levels, subject-independent, 64 EEG channels, 1–45 Hz bandpass filter, topographically mapped time-frequency power features, recurrent-residual NN with custom domain adaptation (Jiménez-Guarneros and Gómez-Gil, 2020).
- Mental arithmetic task: 97.2% accuracy, 2 levels, subject-independent, 23 EEG channels, 0.5–45 Hz bandpass filter, spectral band power features, SVM model (Xiong et al., 2020).
- Visual task: 96% accuracy, 5 levels, subject-independent, 16 EEG channels, 50 Hz notch filter, spectral power band features, classification and regression tree model (Sahin Sadik et al., 2022).

#### 4.2.2 Multitasking

##### 4.2.2.1 Criteria for categorization of studies

To assess the effectiveness of models in multitasking scenarios, we apply similar criteria used in single-tasking studies: classification accuracy, model robustness, and number of MWL levels classified.

However, given the complex nature of multitasking, studies will be categorized based on the task load levels adjustment method and MWL rating (labeling) method. As explained in Section 3.3:

- Based on how task load levels were adjusted, we identified two methods:

° Adding/removing subtasks
° Increasing/decreasing the frequency of occurrences of a constant set of subtasks

- Based on how MWL was rated (labeled), we identified three methods:

° Task load-based
° Subjective MWL-based
° Performance-based

##### 4.2.2.2 Issue of indirect learning: models recognizing subtasks instead of MWL

An important consideration is the risk of models learning to recognize subtasks instead of actual workload levels, which can compromise the validity of MWL classification. Namely, it has been shown that EEG can differentiate between various cognitive activities (Gysels and Celka, 2004; Millan, 2004; Lee and Tan, 2006; Tavakolian et al., 2006; Pušica et al., 2024b). This feature allows ML/DL models to detect the presence or absence of specific subtasks in multitasking scenarios. Consequently, if task load levels are adjusted by adding or omitting subtasks (qualitative task load adjustment), models trained for MWL classification may indirectly learn to recognize subtasks, rather than actual MWL levels. Hence, models may use the learned information about subtasks' presence to deduce about task load (MWL) level.

Obviously, we want to avoid this situation, as we specifically want to estimate MWL levels. However, in the model training phase, there is no way to prevent ML/DL models from inferring MWL levels in this indirect way. The most effective way to address this issue is by focusing on experimental design—specifically, by adjusting task load levels through increasing or decreasing the frequency of occurrences of a constant set of subtasks (quantitative task load adjustment). This way, as the only difference between levels is the frequency of the subtasks, and each level maintains the same set of active subtasks, we can be certain that any variability in EEG is solely due to the actual MWL imposed by the task load. For this reason, we will focus on studies that have followed this experimental design approach (quantitative task load adjustment).

##### 4.2.2.3 Overview of multitasking studies

Despite the fact that there are many different tasks within the multitasking category, a common characteristic they all share is the composition of multiple subtasks, which allows us to analyze them collectively. Some widely used tasks in this category found in the literature include Multi-Attribute Task Battery (MATB) (The multi-attribute task battery for human operator workload and strategic behavior research—NASA Technical Reports Server (NTRS), 1992), Simultaneous Capacity/Multi-Tasking task (SIMKAP) (Bratfisch et al., 2019), driving/flying simulations, systems control simulations, etc.

###### 4.2.2.3.1 MATB

**Task description**

*MATB* is a simulation tool developed by NASA, designed to assess and research human performance and workload in a controlled multitasking environment. The task is designed in a way that it mirrors the demands faced by operators in settings such as aviation and spaceflight. The MATB consists of four subtasks that simulate different activities:

- Tracking: a continuous task where the user must keep a randomly moving target aligned with the center of the screen using a joystick.
- System Monitoring: the user monitors a panel of instruments that occasionally require the user to respond to changes or anomalies by pressing specific buttons.
- Resource Management: a process control task designed to simulate emergencies, allowing for various problem-solving strategies. The user must maintain the liquid levels within specific boundaries by controlling valves. Valves may malfunction, requiring a strategic adjustment in the tank system. Deviations from the designated liquid levels in either tank are considered errors.
- Communications: The user must respond to radio communications by listening for specific call signs and responding by entering appropriate input via the task interface.

The battery is adaptable, allowing researchers to adjust the difficulty of the task to match various study needs.

**Analyzed studies**

(Ke et al. 2023) used a combination of three out of four MATB subtasks—system monitoring, tracking, and resource management to set two task load levels—low and high. In the high-level block, the frequency of the three subtasks was increased compared to the low-level. A rest block was also recorded as a third level. A Support Vector Machine (SVM) model was trained in a session-independent way, classifying EEG segments of 10 s into three classes—rest, low, and high. Results revealed significant classification overlap between low and high levels, with notably poorer accuracies for these levels compared to the rest level and the overall accuracy of 59%. This indicated the model's difficulty in distinguishing between the two levels that varied only in subtask frequencies. Similar conclusions can be drawn from the study of (Salvan et al. 2023), which also used three out of four MATB subtasks in two MWL levels, with frequencies of subtasks varying between the levels. They trained seven ML models with spectral EEG features and found that random forest model achieved the highest accuracy for subject-independent classification (76%). (Pušica et al. 2024b) presented similar problem with distinguishing two highest task load levels that varied only in frequencies of all four MATB subtasks. 50 participants were involved and a CNN model was used to classify 10 s EEG segments, achieving 4-class classification accuracy of 66.2% in session-independent case. Although satisfactory accuracies were obtained for resting level and low level, confusion matrix showed high confusion between medium and high levels. Another study in this category was conducted by (Albuquerque et al. 2022), that used MATB with two difficulty levels, with the same principle applied to the design of the two levels, but with all four subtasks active. A random forest estimator was trained in the subject-independent way. The experimental setup included an interesting additional variation: some participants performed the task running on a treadmill and some performed it cycling on a static bike, introducing physical component. The model classified 4 s EEG segments into the two classes, achieving an accuracy of 70.8% for the treadmill participants and 56.7% for the static cycle participants. This big disparity in accuracies suggests the impact of the physical load on the detectability of MWL through EEG. It is noteworthy, also, that some studies have achieved higher accuracies by configuring task levels within the zone of task overload. For instance, (Hefron et al. 2018) specifically adjusted high task level for each individual, so that the error rate at this level was statistically significantly higher than the rate in the low level. They used MATB with all subtasks active, but adjusted subtasks' frequencies in the high-level block so that individuals, previously trained to the asymptotic performance, make more errors, pushing them toward an overload state. They trained a subject-independent model and achieved accuracies of over 80% for EEG segment lengths of 10, 20, and 30 seconds. Similar results have been reported by (Jin et al. 2024), who designed task difficulty levels so that they were drastically different. Specifically, MATB subtasks in the high-level block have been activated 24 times more frequently than in the low-level block. They also compromised the temporal resolution of MWL predictions by opting for lengthy EEG segments of 3.5 m, which resulted in the highest accuracy (92%). On the other hand, some studies adapted a different approach to designing task load levels. Namely, (Zhou et al. 2023) designed three difficulty blocks by combining different MATB subtasks. In their experiment, the low level involved only the system monitoring and tracking subtasks, the medium level added the resource management subtask, while the hard level had all four subtasks active. This approach made the levels not only quantitatively, but also qualitatively different, as higher blocks had some activities that were not present in the lower levels. For example, hard level engaged participants' auditory capacity with communications subtask, while it was not the case for low and medium levels. As already explained in Section 4.2.2.2, this undermines relevance of the classification results, as models may learn to recognize blocks by detecting specific subtasks, rather than by actual MWL levels. In their study, the model was trained in a subject-independent manner and classified 2 s EEG segments with an accuracy of 75%, using a deep domain adaptation method.

###### 4.2.2.3.2 SIMKAP

**Task description**

*SIMKAP* is a commercial psychological assessment tool developed to evaluate an individual's multitasking capabilities and stress tolerance. Primarily used to screen individuals for roles that demand high multitasking skills, the test is also utilized in various research settings focused on multitasking. The SIMKAP multitasking test involves tasks where participants need to identify and mark identical items across two separate panels, while concurrently, they must answer auditory questions that could involve arithmetic calculations, comparisons, or data lookup. Certain auditory questions are given to be answered later, requiring participants to keep track of time using a clock displayed in the upper right corner. The sequence of questions and tasks is the same for all participants. The studies we analyzed used the public Simultaneous Task EEG Workload Data Set (STEW) (Lim et al., 2018) that involved 48 participants. The dataset comprised 2.5 m. of active task engagement and the resting (baseline) part of each of 48 participants. Upon task completion, participants were asked to separately rate their perceived MWL on a scale of 1 to 9 for both the resting state and active engagement with the task. These subjective rates were taken as the ground truth for MWL models training. Differently from the studies that used MATB and that modified task load levels to impose different MWL levels, here the task load was constant while the perceived MWL, which varied among participants, served as the measure of MWL.

**Analyzed studies**

Raufi and Longo, 2024 applied feature extraction alongside ML models, using subject-independent training, to classify EEG segments into two MWL classes. They mapped the 1–9 self-rated MWL scale into two levels, labeling scores 1–4 as low MWL and 6–9 as high MWL, achieving an accuracy of 90%. However, clustering the dataset this way reduced the MWL classification problem to simply differentiating task condition from the resting condition. Indeed, in the STEW experiment, scores of 1–4 were predominantly assigned to the resting condition, while scores of 6–9 were assigned to the task condition. On the other hand, (Fan et al. 2022) mapped the 1–9 scale into three levels, in the following way: 1–3 labeled as low, 4–6 as medium, and 7–9 as high. They used a recurrent CNN with raw EEG as input to classify EEG segments into the three classes, trained the model in subject-independent way, and achieved an accuracy of 66.8%. (Lim et al. 2018) reported similar results, with high classification accuracy for low MWL class (which mainly included resting condition segments) and big overlapping between medium and high classes. It is worth mentioning that (Fan et al. 2022) also explored training the same model architecture in a subject-dependent manner, achieving a very high classification accuracy (99%). However, this training approach led to serious data leakage issues. Specifically, each participants in the study was involved in just one task block and one resting block, resulting in only one label for the task and one for the resting condition per participant. This is problematic because EEG patterns are known to be distinctive enough to identify individuals (Ma et al., 2015; Valizadeh et al., 2019). So, in subject-dependent classification, where the model is trained and tested on data from the same subject, the model could inadvertently learn to recognize a subject (and the associated MWL rating) based on subject's unique EEG signature, rather than accurately estimate MWL from EEG. This leads to drastically overestimated high classification accuracy. (Yedukondalu and Sharma 2023) also reported high classification accuracy of 4 s EEG segments of 98.1% with the same dataset. However, the training approach they used was random split of segments between training and test sets, raising the same concerns as found in subject-dependent case of (Fan et al. 2022) and undermining the validity of the results. On the other hand, a study by Wang Z. et al. (2024) reported exceptional accuracy for cross-subject robust classification using an attention-based recurrent fuzzy network. Similar to (Fan et al. 2022) they clustered the 1–9 scale into three levels, but achieved a much higher accuracy of 94.4%. Overall, among the papers that focused on the classification of self-assessed MWL levels, this paper stands out with its best-performing model.

###### 4.2.2.3.3 Other tasks

Among the studies that employed other tasks, we highlight two studies that modified levels in a quantitative manner (by increasing or decreasing the frequency of subtasks) but rated (labeled) these levels differently. Firstly, in the experiment by Li H. et al. (2024), participants engaged in an air traffic management task, where the two highest levels (out of four) differed in the number of planes and emergency actions to be managed. The SVM model failed to distinguish between these two levels, leading to a low accuracy of 55%. Secondly, in the experiment by (Fan et al. 2018), participants participated in a simulated driving task with variable driving conditions. Although the conditions objectively varied across levels, the levels were rated and labeled subjectively by a professional. After assigning a rating to each block of the exam, the ratings were categorized into two MWL levels based on a predetermined threshold. Using this approach, the KNN model successfully distinguished between the levels, achieving a high accuracy of 86%. Both studies trained models in a subject-independent way and had quantitatively different task levels. However, the study by (Fan et al. 2018) that rated levels based on subjective ratings achieved much higher accuracy.

##### 4.2.2.4 Summary of multitasking studies

In this Section, we have examined various tasks within the multitasking framework. Our discussion covered the design of tasks, adjustments in task load, alongside assessing different levels of robustness and MWL classification accuracy. We explained the influence of task load adjustments on the validity of MWL classification models. Specifically, we argued that, if task load was adjusted qualitatively, (i.e.,) by adding/removing different subtasks, the model may learn to detect presence of the subtasks, and hence indirectly recognize task level. This practically means that it may have high MWL classification accuracy while not actually learning what was expected of it. That is the reason why, for the purpose of comparison of results across settings, we were interested in the experiments where task load was adjusted quantitatively, (i.e.,) by adjusting frequency of occurrences of subtasks, while they remain active at all levels. We also questioned the validity of results obtained through subject-dependent model training using the SIMKAP (STEW) dataset. Namely, models that were trained and tested with the data of the same subject, may have learned to identify subjects from EEG and recall their MWL rating, thus indirectly predict MWL. This issue arises as there was only one task block and one resting block for each subject and one MWL rating label for each.

Here, we highlight the most relevant solutions based on their robustness and classification accuracy, further categorizing them by the method used for MWL levels rating:

- Task load-based rating of MWL:

° MATB: 76% accuracy, 2 levels, subject-independent, 3 EEG channels, 60 Hz notch filter, spectral band power features, random forest model (Salvan et al., 2023)
° MATB: 66.2% accuracy, 4 levels, session-independent, 24 EEG channels, 1–40 Hz bandpass filter, no features (time series EEG signal), CNN model (Pušica et al., 2024b)
° Air traffic control: 55% accuracy, 4 levels, subject-independent, 59 EEG channels, 0.5–100 Hz bandpass filter, spectral band power features, SVM model (Li H. et al., 2024).

- Subjective rating of MWL:

° SIMKAP: 94.4% accuracy, 3 levels, subject-independent, 14 EEG channel, 1 Hz high-pass filter, spectral band power features, attention-based recurrent fuzzy NN model (Wang Z. et al., 2024)
° Driving simulation: 86% accuracy, 2 levels, subject-independent, 14 EEG channels, 0.2-45 Hz bandpass filter, higher order crossings-based features, KNN model (Fan et al., 2018).

The key takeaway is that studies that used subjective MWL ratings achieved significantly higher accuracy compared to studies that rated MWL based on quantitative task load, that achieved relatively low accuracy. This suggests the high relevance of subjective MWL ratings in complex task settings such as multitasking.

#### 4.2.3 Cross-task MWL classification

Here we examine a group of studies that employed multiple different tasks to evaluate models' performance across the tasks. These studies addressed the problem of cross-task (task-independent) MWL classification, where models are trained on one task and evaluated on different tasks.

##### 4.2.3.1 Overview of cross-task MWL classification studies

Although satisfactory classification accuracies are often achievable within individual tasks, accuracy typically drastically declines when models are applied to cross-task estimation. To date, no universally effective method has been established for satisfactory cross-task MWL estimation. However, some studies reported promising results. For instance, (Zhang et al. 2019), achieved a 89% accuracy across memory and arithmetic tasks. It is important to note, though, that several favorable conditions and task relationships were satisfied. Specifically, their study combined an n-back task with a simple mathematical addition task that incorporated a memory component. The memory component was predominant in the second task, likely allowing the model to focus on this aspect for making predictions. Moreover, the task load levels were closely aligned between the two tasks - NASA-TLX scores indicated roughly equivalent subjective MWL levels for both low and high task loads across the tasks. This means that level one was subjectively perceived as equally demanding in both tasks, and the same holds for level two. Similar consistency was observed in the error rates. This alignment of tasks' characteristics likely contributed to higher cross-task classification accuracy. Furthermore, (Dimitrakopoulos et al. 2017), achieved a binary classification accuracy of 87%, while (Kakkos et al. 2021), reached an even higher accuracy of 94%, both using the same dataset involving an *n*-back task and a mental arithmetic task. In this case, a favorable condition was that the task levels differed significantly in both tasks. This means that level one was significantly less complex than level two in both the first and the second task. Also, level one was rather undemanding in both tasks. Specifically, the n-back task included 0-back and 2-back levels, whereas the mental arithmetic task ranged from additions of 1-digit numbers at the first level to 3-digit numbers at the second level. This setup created highly distinct difficulty levels for each task, thereby enabling the model to learn to distinguish between the levels easier. It would be intriguing to explore the model's performance if the task load levels were closer to each other in terms of difficulty. Another case where cross-task estimation has shown promising results is when the tasks were highly similar. For example, (Guan et al. 2023), utilized four types of *n*-back tasks: verbal, object, space (verbal), space (object), each with three levels (*n*-values from 1 to 3), achieving an accuracy of 81.3%. A key factor here was that all four tasks were memory tasks of the same format, differing only in the types of items to be memorized. However, in most cases where the tasks were sufficiently distinct, cross-task MWL estimation tended to be unsuccessful. For example, (Baldwin and Penaranda 2012), tested cross-task estimation among three similar but distinct-enough memory tasks, achieving satisfactory within-task classification accuracies of 86–89%, but cross-task accuracy was below chance level. Similarly, (Hernández-Sabaté et al. 2022), trained their model on a hybrid n-back task with three levels and evaluated it on a flight simulation task, finding that MWL predictions did not correlate with the actual difficulty levels of the flight simulation task.

##### 4.2.3.2 Summary of cross-task MWL classification studies

Cross-task MWL estimation, to date, has not proven universally feasible, though it has shown some promising results under certain conditions. As we have seen, it tends to be more successful when tasks are more similar and when the MWL levels across those tasks are aligned in terms of difficulty. This suggests that when tasks share common cognitive demands and similar intensity levels, it is easier for the models to generalize MWL estimation from one task to another. However, in scenarios where the tasks are different-enough, model performance across such varied conditions is compromised. This is a critical limitation in the current state-of-the-art MWL estimation methods, emphasizing the challenge of developing models that are robust and adaptable across a diverse range of task types.

#### 4.2.4 Summarizing MWL classification across task categories

We evaluated MWL classification studies across different task categories, emphasizing the distinction between single-tasking and multitasking settings. By exploring these categories and their subcategories, this analysis examined how the task setting influences EEG-based MWL estimation using machine learning. We discussed the way MWL classification models were trained, discussed validity and interpreted the results. Particularly, we focused on:

- *At least cross-session robust* models—for the most indicative and relevant results, considering data leakage issues (as explained in Section 4.1).
- *MWL levels within cognitive capacities*—where individuals could handle the given task load.

Therefore, in each task category, the accuracies of the top-performing models that satisfied the above-defined conditions were taken as representatives of the corresponding categories. However, the number of classes (MWL levels) varied across studies, influencing model accuracies, which needs to be taken into account when comparing the performances of different models. For example, a model classifying into five classes that achieves the same accuracy as one classifying into two classes should be graded higher due to its greater granularity.

To address this challenge of comparing models with different accuracies and numbers of classes, we adopted Cohen's Kappa as a more informative metric (Cohen, 1960). Cohen's Kappa measures the agreement between two classifiers (or in this context, the agreement between the predictions and the actual labels). It is calculated using the formula:


$$\begin{array}{l} \kappa =\frac{p_{0}-p_{e}}{1-p_{e}} \end{array}$$

Where:

- *p*_0_ (in this context) is the accuracy of the classifier
- *p*_*e*_ is the chance-level accuracy (expected accuracy of a random classifier considering labels distribution)

Values of κ range from −1 to 1: negative values indicate less than chance-level accuracy, value 0 indicates chance-level, while value 1 indicates perfect accuracy. This metric thus provides a measure of classifier performance that factors in the granularity of classification, by penalizing classifiers that may have higher accuracy due to lower number of classes.

The summary of the performance comparison procedure is as follows:

1. Categorization of studies based on (see Section 3.3):

a. Task type and subtype
b. MWL rating (labeling) method
c. Task load adjustment method (for multitasking)

2. Filtering of studies to include only those that:

a. Performed at least session-independent evaluation
b. Ensured MWL levels remained within cognitive limits of participants
c. Did not use qualitative task load adjustment (for multitasking), as explained in Section 4.2.2.2

3. Standardization of performance metric using Cohen's Kappa, to account for differing numbers of MWL classes
4. Identification of best-performing models within each task category (as defined in step 1), based on Cohen's Kappa
5. Comparison of model performance across task categories

**Figure 10:**
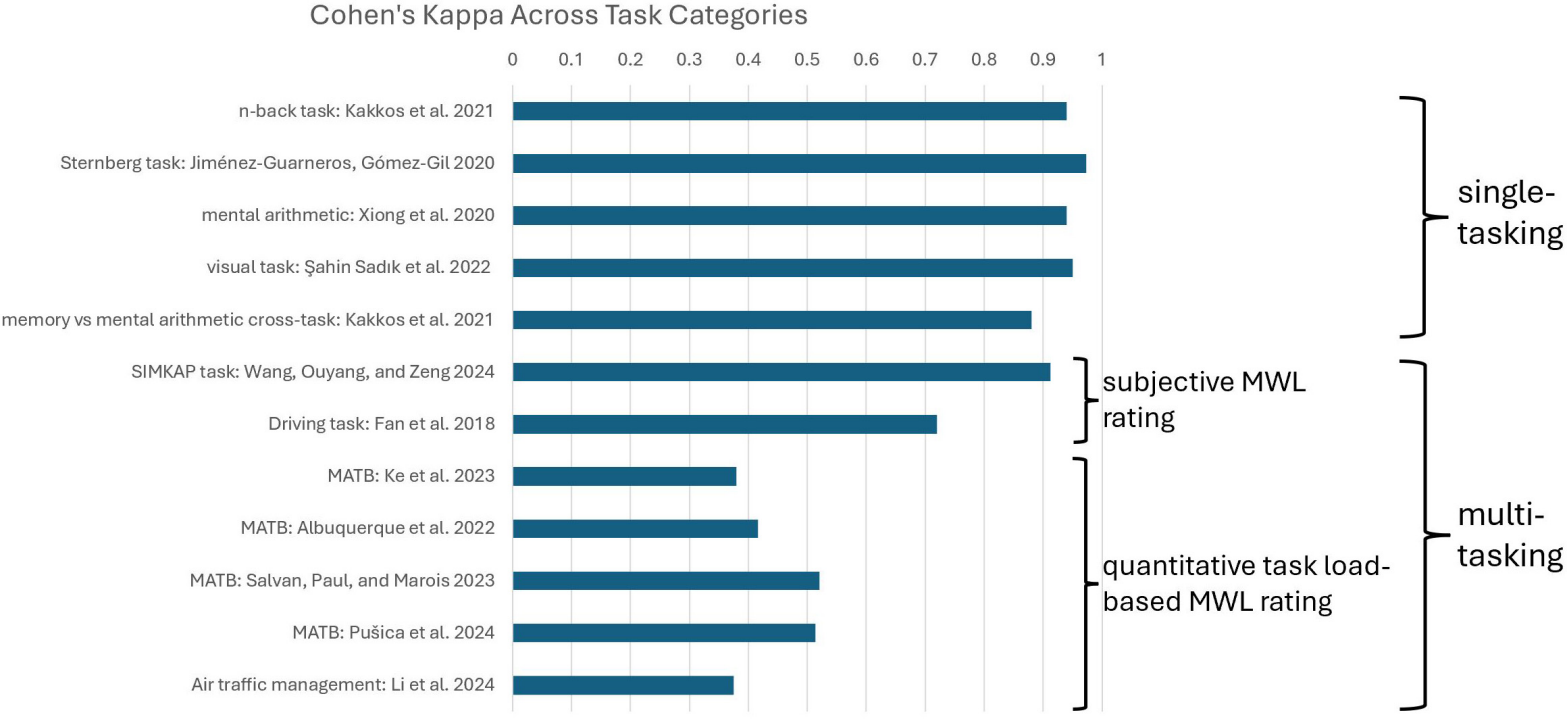
Highest Cohen's Kappa scores (classification metric) across task categories.

Results of this analysis are shown in Figure 10, where Cohen's Kappa of the best-performing models across task categories are shown. A significant observation from this analysis is the noticeable drop in MWL classification performance reported in multitasking studies where MWL was rated based on quantitative task load.

As can be observed from the figure, there is a substantial Cohen's Kappa gap between best performing models in multitasking studies employing task load-based MWL ratings and best performing models in single-tasking and studies employing subjective MWL ratings.

### 4.3 Experimental design flaws in the literature: insights and recommendations

In this section, we systematized the experimental design flaws identified in the analyzed literature, providing corresponding recommendations. The following flaws were commonly identified across multiple papers:

- Lack of transparency: Sufficient details about the design of MWL levels, the length of EEG segments used as inputs to models, explanation of train/test data split, and error rates (participants' performance information) were omitted in some papers.
- Data leakage: Randomly splitting EEG segments between training and testing sets results in data leakage, as explained in Section 4.1. To avoid this, experimental sessions (or separate task blocks, depending on the experimental design) should not be divided between the train and test sets - all segments from the same session/block should be assigned to the same set (either train or test). This ensures session-independent estimation (note that subject-independent estimation is inherently session-independent as well). Data leakage leads to drastically overestimated model performance, which is misleading when the model is applied to different sessions or subjects not included in the training set.
- Qualitatively different MWL levels: In multitasking experiments with different sets of subtasks or single-tasking experiments with different tasks across MWL levels, models may learn to detect task-related EEG patterns rather than MWL-related patterns, as explained in Section 4.2.2.2. This causes the models to classify MWL levels indirectly, based on task-related patterns. To address this issue, the same tasks/subtasks should be used across all MWL levels.
- Too large differences between MWL levels: This issue often arises when there are only two MWL levels and one of them is either baseline condition (passive state) or is significantly easier than the other. In such cases, classification performance metrics tend to be high because levels with significant differences are easier to differentiate. A good way to address this issue is to design multiple MWL levels in the experiment, which increases estimation resolution and contributes to the model's applicability.
- Overload MWL level: When participants make errors due to high task load, their actual MWL level cannot be reliably determined. If the task load exceeds their capacity and they are unable to handle the task, their MWL may not increase proportionally. In such cases, participants may experience low MWL despite a high task load, leading to misleading interpretations and incorrect dataset labeling. To address this issue, task error rates (performance on the task) should be monitored, and MWL levels could be individually adjusted for each participant based on their performance.
- Lack of subjective evaluations: Incorporating subjective evaluation methods, such as questionnaires, into experiments can provide valuable insights into participants' perceived MWL. This is particularly important because subjective MWL levels often differ significantly from objective task load measures (Pušica et al., 2024b). Furthermore, models trained on subjective MWL levels have shown promising results in multitasking scenarios (Wang Z. et al., 2024; Fan et al., 2018), where task load-based labeling was inadequate. Integrating subjective evaluations into experimental designs offers a more comprehensive understanding of MWL and enables more accurate data labeling.

Finally, as a critical issue, we wanted to highlight the absence of standardized protocols and guidelines for designing experiments and the lack of established benchmark datasets for evaluating MWL estimation models' performance. This makes the comparison of results across studies challenging. Therefore, developing guidelines and protocol for designing experiments and benchmark datasets would be highly beneficial for the research community. These standardized frameworks would need to include a diverse range of task types that engage different cognitive resources. Such diversity would also enable the evaluation of MWL estimation solutions across tasks, addressing generalized (task-independent) MWL estimation as one of the most challenging and significant problems in the field. Moreover, benchmark datasets would reduce the time and effort required for experimental design, data collection, and processing, providing researchers with a common foundation to build upon.

## 5 Conclusions

In this review we systematically explored the performance of machine learning models for EEG-based MWL classification across different task types and experimental settings.

We focused specifically on machine learning models that exhibited at least session-independent level of robustness - to avoid falsely optimistic accuracies that can result from data leakage. We also concentrated on setups where MWL remained within the cognitive capacity of participants, (i.e.,) where they could handle the given task load. Additionally, when analyzing multitasking scenarios, we focused on cases where MWL levels were designed by adjusting frequencies of subtasks rather than by changing sets of subtasks across the levels (quantitative MWL adjustment). This was to avoid the risk of models merely learning to detect specific subtasks and hence indirectly predict MWL levels, rather than genuinely learning to estimate MWL, as explained in Section 4.2.2.2.

A key finding of this review highlights the challenge of task load-based MWL estimation (used in the vast majority of studies) in more complex task categories such as multitasking. This is particularly relevant for practical applications, as real-world tasks typically involve at least some degree of multitasking. This conclusion is supported by the significant drop in classification accuracy observed in multitasking studies that employed task load—based MWL ratings. The drop was evident when compared to the best-performing ML/DL models in single-tasking studies and those using subjective MWL ratings (Figure 10).

An important limitation of this review is the scarcity of studies that meet the previously outlined criteria. Therefore, more studies in this domain are necessary to enhance the reliability of conclusions. Another limitation is the variability in MWL levels design across studies. Namely, MWL classification accuracy is significantly influenced by how distinctly the MWL levels are designed in experiment—when they are more different, higher accuracy is expected as the models have a wider margin for estimation. This complicates the comparison of models' performances across different experiments.

## Data Availability

The original contributions presented in the study are included in the article/supplementary material, further inquiries can be directed to the corresponding author.
